# Regulation of *Neisseria meningitidis* cytochrome *bc*_1_ components by NrrF, a Fur‐controlled small noncoding RNA

**DOI:** 10.1002/2211-5463.12266

**Published:** 2017-08-05

**Authors:** Yvonne Pannekoek, Robert Huis in ‘t Veld, Kim Schipper, Sandra Bovenkerk, Gertjan Kramer, Dave Speijer, Arie van der Ende

**Affiliations:** ^1^ Department of Medical Microbiology Center for Infection and Immunity Amsterdam (CINIMA) Academic Medical Center The Netherlands; ^2^ Department of Medical Biochemistry Academic Medical Center Amsterdam The Netherlands; ^3^ Present address: Genome Biology Unit EMBL Heidelberg Heidelberg Germany

**Keywords:** cytochrome *bc*_1_, *Neisseria meningitidis*, NrrF, riboregulation, sRNA

## Abstract

NrrF is a small regulatory RNA of the human pathogen *Neisseria meningitidis*. NrrF was previously shown to repress succinate dehydrogenase (*sdhCDAB*) under control of the ferric uptake regulator (Fur). Here, we provide evidence that cytochrome *bc*_*1*_, encoded by the polycistronic mRNA *petABC*, is a NrrF target as well. We demonstrated differential expression of cytochrome *bc*_*1*_ comparing wild‐type meningococci and meningococci expressing NrrF when sufficient iron is available. Using a *gfp*‐reporter system monitoring translational control and target recognition of sRNA in *Escherichia coli*, we show that interaction between NrrF and the 5′ untranslated region of the *petABC* mRNA results in its repression. The NrrF region essential for repression of *petABC* was identified by site‐directed mutagenesis and is fully conserved among meningococci. Our results provide further insights into the mechanism by which Fur controls essential components of the *N. meningitidis* respiratory chain. Adaptation of cytochrome *bc*_*1*_ complex component levels upon iron limitation is post‐transcriptionally regulated via the small regulatory RNA NrrF.

**Abbreviations**
BIGSdb: Bacterial Isolate Genome Sequence database
Fur: ferric uptake regulator
RBS: ribosome binding site
SD: Shine–Dalgarno
Spp: Species
WTA: whole transcriptome analysis
wt: wild‐type

Riboregulated networks in which small RNA (sRNA) regulate both stability and accessibility (and thus, translation) of transcripts (mRNA) abound in prokaryotes. Hundreds of sRNA have been identified by using a wide variety of both computational and experimental approaches in various bacterial species during the last decade. One class of sRNA, typically ranging in size between 50 and 300 nucleotides, is found in intergenic regions distant from the genes encoding their mRNA targets. With few exceptions, they do not contain open reading frames and typically have limited regions of complementarity (10–30 nucleotides), displaying imperfect base pairing interactions with their mRNA targets 1, 2, 3.

Detailed studies of these so‐called *trans*‐encoded sRNA have revealed general principles in their mode of action. The majority of these sRNA interact by noncontiguous base pairing with mRNA targets by an antisense mechanism to activate or, more frequently, to repress translation of target mRNA. Duplex formation generally results in sequestering of the Shine–Dalgarno (SD) or translational start codon (AUG) sequences of the ribosome binding site (RBS). This duplex formation will compete with initiating ribosomes and will lead to ribosome occlusion, often followed by rapid RNA decay. This model of sRNA action is supported by many examples of sRNA that pair around the SD or AUG to inhibit translation 4, 5, 6, 7, 8, 9, 10. In numerous, mostly Gram‐negative bacteria, the RNA‐binding protein Hfq is required for the action and stability of many *trans*‐encoded sRNA 11. Thus, translational regulation can be achieved by a variety of mechanisms. Translation can be repressed via catalytic degradation of mRNA: through coupled degradation of sRNA·mRNA duplexes or through mRNA sequestering, or activated by opening up the ribosome binding site 2, 12, 13. In many cases, the aforementioned RNA chaperone Hfq mediates the interactions between *trans*‐encoded sRNA and their mRNA targets and protects the sRNA from RNase E‐mediated degradation 5, 11, 14, 15. Environmental stimuli, including anaerobic conditions, oxidative stress, glucose availability, osmotic imbalance and iron availability, may affect the expression of sRNA 12, 16, 17.

In the opportunistic strictly human pathogen *Neisseria meningitidis,* several *trans*‐encoded sRNA have been described. AniS is anaerobically induced through activation of its promoter by the Fnr global regulator and downregulates the expression of a protein of unknown function 18, 19. Recently, we identified sibling sRNA targeting genes encoding TCA cycle enzymes stressing their importance in the adaption to changing environments of the host. The riboregulated network of the sibling sRNA is part of the RelA‐regulated stringent response 20. NrrF is synthesized under iron‐limiting conditions and controlled by the ferric uptake regulator (Fur) 21, 22. Fur is essential for iron homeostasis in many prokaryotes 23. Fur inhibits expression of genes essential for iron acquisition by means of binding to a specific consensus target sequence (the so‐called Fur box) in their promoters. Fur also acts as a positive regulator, affecting the production of factors storing or containing iron 24. The succinate dehydrogenase complex, encoded by the polycistronic operon *sdhCDAB,* donates electrons to cytochrome *bc*_1_ in the respiratory electron transport chain 25. Its expression is regulated by iron and Fur, but the promoter of *sdhCDAB* lacks a Fur box 19, 26, 27. Instead, *sdhCDAB* was shown to be regulated by NrrF 21, 22, demonstrating crosstalk between the riboregulated network and the Fur‐regulated network in meningococci. By using a well‐established heterologous (*Escherichia coli*) reporter system for translational control and target recognition of sRNA *in vivo,* we showed a direct interaction between NmsR‐A and *sdhC* 20, 28; though, we did not observe regulation in meningococci. The regulation of *sdhC* by multiple sRNA has been shown before, and *sdhCDAB* might thus be the first example of a cistronic mRNA in meningococci that is subjected to regulation by two different sRNA species 29. The cytochrome *bc*_1_ complex transfers electrons to c‐type cytochromes *c*_2_, *c*_4_ and *c*_5_, which in turn donate electrons to the cytochrome *cbb3* complex. Cytochrome *c*_5_ is also an important electron donor for the AniA nitrite reductase 30, 31, 32. Expression of the cytochrome *bc*_1_ complex, the cytochromes *c*_4_, *c*_5_ and the cytochrome *cbb*_3_ complex also appears to be controlled by Fur and/or iron but the promoter regions of the genes encoding these proteins lack a Fur box as well 19, 26, 27. Hence, the mechanism by which the expression of these genes is controlled is unknown.

We hypothesized that more components of the respiratory electron transport chain of meningococci that are under indirect control of Fur could be subjected to regulation by sRNA and studied the potential role of NrrF in this regulation. Here, we focused on regulation of the cytochrome *bc*_1_ complex, encoded by the polycistronic operon *petABC*. Differential protein profiling of a *nrrF* mutant *vs*. meningococci overexpressing *nrrF* showed downregulation of PetA. This downregulation was confirmed by western blotting. Direct interaction between NrrF and the 5′ untranslated region (5′UTR) of *petABC* was again confirmed in a heterologous *gfp*‐reporter system 28.

Thus, we first show that NrrF is involved in the regulation of proteins involved in iron uptake, iron‐dependent metabolic processes and the oxidative stress response. We then further characterize a novel target of NrrF, functionally involved in respiration in *N. meningitidis,* extending the experimentally validated NrrF‐regulated network of *N. meningitidis*. In addition, we provide important insights into the mechanism by which an essential component of the respiratory chain is indirectly controlled by Fur. Adaptation of expression of components of the cytochrome *bc*_1_ complex to iron limitation is mediated at the post‐transcriptional level through the action of the small regulatory RNA NrrF.

## Materials and methods

### Bacterial strains and culture conditions

All strains used in this study are listed in Table 1. *Neisseria meningitidis* strain H44/76, B: P1.7,16: F3‐3: ST‐32 (cc32), is closely related to the serogroup B strain MC58, belonging to the same clonal complex 33, 34. Meningococci were cultured in GC broth or on GC plates supplemented with 1% (v/v) Vitox (Difco/Oxoid, Thermo Fisher Scientific, Waltham, MA, USA) at 37 °C in a humidified atmosphere of 5% CO_2_. Plates or broth were supplemented with kanamycin (Km) (100 μg·mL^−1^), erythromycin (Erm) (5 μg·mL^−1^) and/or chloramphenicol (Cm) (5 μg·mL^−1^). Overexpression of *nrrF* in meningococci was induced after 4 h of growth in liquid culture of strains containing 3XFLAG‐tagged genes by the addition of 0.5 mm IPTG to the culture medium for 1 h. Growth in broth was monitored by measuring optical density of cultures at 530 nm (OD_530_) at regular time intervals. *Escherichia coli* strain Top10 (Invitrogen, Thermo Fisher Scientific, Waltham, MA, USA) was used to clone *gfp* fusions, and in experiments that involved co‐expression of *gfp* fusions and sRNA. *Escherichia coli* strain Top10F' (Invitrogen, Thermo Fisher Scientific) was used to clone sRNA expression plasmids. *Escherichia coli* strains were grown in lysogeny broth (LB) or on LB plates at 37 °C. Antibiotics were applied at the following concentrations: 100 μg·mL^−1^ ampicillin and 20 μg·mL^−1^ chloramphenicol. *Neisseria meningitidis* H44/76 *hfq‐*knockout mutant (∆*hfq*) was constructed as described previously 35.

**Table 1:** Plasmids and strains used in this study

Plasmids			
Name	Plasmid backbone	Genotype or characteristics	References
		Target gene^a^ (Gene id^b^)	
pXG‐0	pZA31‐luc	Control for cellular autofluorescence	28
pXG‐1	pZA31‐luc/pXG‐10	Control for sRNA effect on *gfp* expression	28
pXG‐10	pXG‐0/pWH601	for construction of *gfp* fusions	28
pPetA::gfp	pXG‐10	*petA* (NMB2053)	This study; 28
pNmNrrF	pZE12‐luc	*nrrF* (IGR NMB2073‐NMB2074)	This study; 28
pJV300	pZE12‐luc	Control nonsense RNA	28
pEN11_NrrF	pEN11_pldA	*nrrF* (IGR NMB2073‐NMB2074)	This study; 75
pEN11_Empty	pEN11_pldA	*Vector control*	This study; 75
pDOC‐F	pEX100T	3XFLAG::Km^R^	39
pCR^®^2.1			Invitrogen

Strains		
Name	Relevant marker/Genotype	References
*Neisseria meningitidis*		
H44/76	Parental strain	33, 34
H44/76Δ*nrrF*	*nrrF* knockout	This study
YPS1004	*petA*::FLAG::Km^R^	This study
YPS1006	∆*hfq*::Erm^R^ *petA*::FLAG::Km^R^	This study
*Escherichia coli*		
Top10	*mcr*A ∆ *(mrr‐hsd*RMS*‐mcr*BC) ∆8*0lac*Z∆M15 ∆*lac*X74 *deo*R *rec*A1 *ara*D139 ∆ *(ara‐leu*)7697 *gal*U *gal*K *rps*L *end*A1 *nup*G	Invitrogen
Top10F'	F′[*lacIq* Tn10 (*TetR*)] *mcr*A ∆ *(mrr‐hsd*RMS*‐mcr*BC) ∆8*0lac*Z∆M15 ∆*lac*X74 *deo*R *rec*A1 *ara*D139 ∆ *(araleu*)7697 *gal*U *gal*K *rps*L *end*A1 *nup*G	Invitrogen
JVS‐2001	∆*hfq*::Km^R^	28

a Gene whose N‐terminal coding sequence was fused to *gfp*.

b Gene ID or location, according to the annotation of the genome of strain MC58 36.

### Plasmids and oligonucleotides

All plasmids used in this study are listed in Table 1 and oligonucleotides in Table S1. Plasmid pCR^®^2.1 (Invitrogen) was used for cloning and sequencing of PCR products. Plasmids pXG‐0 (control for autofluorescence), pXG‐1 (control for sRNA effect on *gfp* expression) and pXG‐10 (standard plasmid for *gfp* fusion cloning) were kindly provided by J. Vogel (Würzburg) and have been described previously 28. The 5′UTR of *petA* was fused with *gfp* in pXG‐10, thereby creating *petA::gfp* using primer pair ALpetAF/ALpetAR. The *nrrF* gene was inserted into the sRNA‐expressing plasmid based on pZE12‐*luc*, containing a ColE1 replicon and a strong *rrnB* terminator, thereby creating pNmNrrF using primer pair ALsRNA3F/ALsRNA3R2. Cloning strategies used are described in 28. The *nrrF* gene of *N. meningitidis* strain H44/76 is 100% identical to the sequence of the gene as present in strain MC58 34, 36. It was cloned at the transcriptional start and end points, as determined by whole transcriptome analysis (WTA) 37. Mutations were generated using Quickchange site‐directed mutagenesis (Stratagene, La Jolla, CA, USA). A *nrrF*‐knockout mutant (Δ*nrrF*) was created by homologous recombination, replacing *nrrF* with a Erm resistance cassette ligated between PCR products of primer pairs YPsRNA3FWKO1/YPsRNA3RPKO2 and YPsRNA3FWKO3/YPsRNA3RPKO4 as described before 35. Correct insertion and orientation of *Erm* was confirmed with Sanger sequencing using primer pairs YPsRNA3FWKO1/JP22 and YPsRNA3FWKO4/JP19. Overexpression of NrrF in meningococci was achieved using the cloning strategy as described previously 38. In brief, to construct a vector allowing overexpression of *nrrF* in meningococci (designated pEN11_NrrF), the region from the unique MauBI through one nucleotide upstream from the transcriptional start of shuttle vector pEN11_*pldA* was PCR‐amplified using primer set YPpen11MauBI/YPpen11plus1. The *nrrF* gene was amplified using primer set RH_NrrF_C_FW1/RH_NrrF_C_RV1. Both fragments were ligated and PCR‐amplified using YPpen11MauBI/RH_NrrF_C_RV1 as primer combination. The resulting PCR product was digested with MauBI and BspHI and ligated into the ~8600‐bp gel‐purified MauBI and BspHI‐predigested pEN11 core plasmid. Appropriate clones were selected using primer pair pen11FW2/pen11R. Expression of *nrrF* upon induction was verified by RT‐qPCR. Plasmid pDOC‐F was used as template to generate a DNA fragment by PCR coding for 3xFLAG and Km resistance 39. A vector control strain was created by cloning pEN11 with *pldA* removed in the *nrrF‐*knockout strain (Δ*nrrF*+pEN11_Empty). All constructs were verified by Sanger sequencing.

### Differential protein profiling using LC‐MS^E^

*Neisseria meningitidis* H44/76Δ*nrrF*+pEN11_Empty and H44/76Δ*nrrF*+pEN11_*nrrF* (three biological replicates each) were grown in iron‐rich broth (see above) to logarithmic phase (OD_530_~0.5), centrifuged and frozen at −80 °C. Subsequently, reverse‐phase liquid chromatography followed by data‐independent alternate scanning mass spectrometry (LC‐MS^E^) was performed as described 40, 41. Differential expression was analysed by Student's *t*‐test (two‐tailed distribution, equal variances assumed) at *P* ≤ 0.01 with false discovery rate (FDR) control according to Benjamini–Hochberg (BH) at *q* ≤ 0.05 42. When appropriate, phase‐variable genes were discarded 36, 43, 44, 45, 46, 47, 48, 49. To allow for statistical analysis of proteins detected in only one condition, proteins in other samples were assumed to be quantified at least 10% lower than the lowest detected protein (0.08). Up to two samples were given the value 0.07 to minimize overestimation of significance and fold regulation. Previously, this approach led to results that could be satisfactorily confirmed by independent experiments 41. Gene identification was taken from the original *N. meningitidis* MC58 annotation and updated using the KEGG and UniProt databases 36, 50, 51.

### Construction of a meningococcal strain expressing a C‐terminal 3XFLAG‐tagged cytochrome *bc*_*1*_

A meningococcal strain coding for 3XFLAG‐tagged cytochrome *bc*_1_ was constructed as follows. First, a fragment of ~500 bp, ending three nucleotides upstream of the translational STOP codon of *petABC*, was amplified using the primer pair KSpetC1/KSpetC2 and genomic DNA of strain H44/76 as template to generate fragment A. Next, the 3xFLAG tag and the Km resistance cassette were amplified using plasmid pDOC‐F as template and primer set pDOCF1 and pDOCF2 to generate fragment B. Last, a ~500‐bp fragment downstream of the STOP codon of the gene to be 3XFLAG‐tagged was amplified using primer pair KSpetC3/KSpetC4 and genomic DNA of strain H44/76 as template to generate fragment C. Fragment A was ligated to fragment B and the ligation product was amplified using primer KSpetC1/KSpDOCF2 to generate fragment AB. Next fragment C was ligated to fragment AB and amplified using primer KSpetC1/KSpetC4 to generate fragment ABC. The 3XFLAG tag at the C terminus of *petABC* was introduced via homologous recombination into the chromosome of strain H44/76 or ∆*hfq* by transformation of the DNA fragment and selection of transformants for Km resistance. *Neisseria meningitidis* was transformed as described previously 52. Resistant transformants were checked by PCR and Sanger sequencing for integration and orientation of the FLAG epitope and the Km resistance cassette.

### Western blotting

SDS/PAGE and western blotting were performed as previously described 53. Briefly, culture samples of 1 mL were taken and centrifuged for 3 min at 16 000 ***g***. Pellets were resuspended in 1x SDS/PAGE loading buffer (83 mm Tris/HCl pH 6.8, 13% β‐mercaptoethanol, 2% SDS, 3% glycerol, 0.01% bromophenol blue) in such a volume that all samples contain an equal number of cells that is approximately equivalent to an OD_600_ of 0.01 per μL. Then, samples were heated for 10 min at 99 °C and an equivalent of an OD_600_ of 0.1 was loaded onto an 11% SDS/PAGE. Proteins of the whole‐cell fractions were separated at 30 mA for 4 to 5 h. Gels were transferred overnight to nitrocellulose (Schleicher & Schuell BioScience, Dassel, Germany) at 50 mA in a wet tank using a bicarbonate buffer (10 mm NaHC0_3_, 3 mm Na_2_CO_3_ pH 9.9) containing 20% (v/v) methanol as transfer buffer. Blots were blocked for 15 min at room temperature (RT) with western buffer (10 mm Tris/HCl, 500 mm NaCl, 0.5% (v/v) Tween‐20, pH 7.5) followed by incubation for 1 h at RT with anti‐FLAG monoclonal (Sigma‐Aldrich, Altstadt, Germany) in the same buffer. Then, blots were rinsed for 15 min with western buffer, 1 min with tap water and incubated with the second antibody, anti‐mouse alkaline phosphatase (Promega, Madison, WI, USA) (1 h RT) in western buffer. Next, blots were washed and rinsed and developed using 66 μL nitroblue tetrazolium chloride (NBT) (50 mg·mL^−1^) (Promega) and 33 μL (50 mg·mL^−1^) of 5‐bromo‐4‐chloro‐3′‐indolyl phosphate p‐toluidine salt (BCIP) (Promega) was added to 10 mL developer buffer (0.1 m Tris/HCl, 0.1 m NaCl, 5 mm MgCl_2_ pH 9.5). Quantification of western blot images was performed using Gene Tools Image analysis software (Syngene, Frederick, MA, USA). Western blots were repeated five times. The results of a representative experiment are shown.

### RNA isolation and RT‐qPCR

RNA was extracted from meningococci grown to log phase (OD_600_ 0.2–0.5) using the miRNeasy mini kit (Qiagen, Hilden, Germany) followed by Turbo DNAse TURBO DNA‐*free™* kit (Life Technologies, Thermo Fisher Scientific) treatment. Then, cDNA was synthesized from 1.5 μg of RNA and random oligonucleotide hexamers using ThermoScript™ RT (Invitrogen, Thermo Fisher Scientific) according to the manufacturer's recommendations. Quantitative PCR was performed using LightCycler^®^ 480 SYBR Green I Master in the LightCycler^®^ 480 System (Roche, Basel, Switzerland). Identities of the resulting amplicons were checked by melting curve analysis using the LightCycler 480. Reaction mixtures containing no template were included in each real‐time PCR experiment to control for contamination. Transcripts of target and reference genes were analysed using LinRegPCR version 2014.2 54. Constitutive relative gene expression in media was determined as a ratio of target gene *vs*. reference genes [*rmpM* (NMB0382) and *cbbA* (NMB1869)].

### Liquid culture whole‐cell fluorescence measurements and data processing

*Escherichia coli* Top10 cells expressing *petA*::*gfp* fusions were streaked on standard LB plates supplemented with appropriate antibiotics. After overnight growth, colonies were photographed in a Syngene bioimage analyser (Syngene, Frederick, MA, USA) using a Lumenera (Ottawa, ON, Canada) camera with a 510‐nm emission filter and a 460‐nm excitation filter. Fluorescence measurements in 96‐well plates were taken as described 28. In brief, single colonies (in triplicate) of *E. coli* strains harbouring a *petA*::*gfp* fusion and sRNA‐expressing plasmids were inoculated in 200 μL LB in a 96‐well microtitre plate and cultures were grown at 37 °C. The OD was measured at 620 nm in a ELISA reader (Anthos Labtec, Salzburg, Austria) and fluorescence was measured (optical excitation filter 485/420 nm, emission filter 530/525 nm) in a Cytofluor II multiwell plate reader (PerSeptive Biosystems, Framingham, MA, USA). The linear range of increasing fluorescence during growth covered by all members of a triplicate was selected to obtain the specific fluorescence. To calculate the specific fluorescence, the total fluorescence of a given strain expressing NrrF and *petA*::*gfp* (the mean fluorescence of the triplicate at a chosen time point within the linear range) was corrected for the autofluorescence measured in strains harbouring the NrrF expression plasmid (pNmNrrF) or control nonsense sRNA (pJV300) in combination with the negative control plasmid pXG‐0 [expressing luciferase (*luc*), *i.e*. no *gfp*]. The regulatory effect of NrrF on the *petA::gfp* fusion was expressed as fold regulation (mean of the triplicate values). This is calculated by dividing the unregulated *petA::gfp*‐specific fluorescence (negative control sRNA (pJV300) by the regulated specific fluorescence *petA::gfp* [sRNA of interest (pNmNrrF)].

## Results

### Proteomic analysis of *Neisseria meningitidis nrrF* mutant vs. overexpression strains shows changes in proteins involved in iron homeostasis and oxidative stress response

A total of 559 proteins were reliably detected in the *nrrF* mutant and overexpression mutants, representing a quarter of the predicted protein‐coding content of *N. meningitidis* strain MC58 36 (Table S2). Twenty‐three proteins were differentially regulated at *P* ≤ 0.01, 13 being upregulated and 10 downregulated (Table 2). NrrF is a Fur‐induced sRNA and is typically repressed in an iron‐rich environment. We hypothesized that artificially overexpressing *nrrF* in an iron‐rich medium would upregulate the translation of proteins normally involved in iron uptake and downregulate proteins involved in iron‐dependent processes. Indeed, three proteins involved in iron transport (ExbB and FbpA) and (host‐acquired) haeme oxidation (HemO) are upregulated. Among the downregulated proteins are CyaY, a protein involved in iron‐sulfur‐cluster protein assembly and the oxidative stress response protein glutathione peroxidase (GpxA). Of special interest, two other proteins involved in iron‐dependent energy generating respiration are downregulated as well. They are the previously identified target SdhA and a novel putative target of NrrF, PetA.

**Table 2:** Overview of genes differentially regulated in Δ*nrrf*+pEN11_Empty vs. Δ*nrrf*+pEN11_*nrrf* (*P ≤* 0.01)

Gene ID^a^	Name^a^	Function^b^	Pathway or Biological role^b^	Fold change^c^	*In silico* target^d^
*Upregulated*					
NMB2096	*mqo*	Malate:quinone oxidoreductase	TCA cycle	**10.1**	
NMB2086	*obg*	GTPase	GTPase	8.7	
NMB0378	*cysP*	Putative phosphate permease	Ion transporter	8.6	
NMB1839	*fhs*	Formate‐tetrahydrofolate ligase	Carbon metabolism	7.2	
NMB0477		GTP‐binding protein	Unknown	5.1	Yes
NMB1524		FAD‐binding oxidoreductase	Unknown	3.4	
NMB1383	*hscB*	Co‐chaperone protein	Protein folding	**3.2**	
NMB1428		Putative metalloaminopeptidase	Aminopeptidase	2.6	
NMB1729	*exbB*	Biopolymer transport protein	Iron transporter	2.0	Yes
NMB1445	*recA*	Protein RecA	DNA replication & repair	1.9	
NMB1946		Outer membrane lipoprotein	Unknown	1.2	
NMB0634	*fbpA*	Major ferric iron binding protein	Iron transporter	1.2	
NMB1669	*hemO*	Heme oxygenase	Iron oxidation	**1.2**	
*Downregulated*					
NMB1710	*gdhA*	Glutamate dehydrogenase	Amino acid metabolism		
NMB0479		Uncharacterized protein	Unknown	−1.6	
NMB0171	*minD*	Septum site‐determining protein	Cell division	−2.1	Yes
NMB1978	*cyaY*	Protein CyaY	Fe‐S cluster assembly	−2.6	
NMB2053	*petA*	Ubiquinol‐cytochrome c reductase iron‐sulfur subunit	Oxidative phosphorylation	−4.8	Yes
NMB0015	*gnd*	6‐phosphogluconate dehydrogenase	Pentose phosphate pathway	−7.3	
NMB0950	*sdhA* ^E^	Succinate dehydrogenase flavoprotein subunit	TCA cycle	−8.2	Yes
NMB1347	*suhB*	Extragenic suppressor protein	Carbohydrate metabolism	−**9.3**	
NMB0933	*tadA*	tRNA‐specific adenosine deaminase	Transcription/translation	−**13.3**	
NMB1621	*gpxA*	Glutathione peroxidase	Oxidative stress	−**13.7**	Yes

a Gene identification and name according to original MC58 annotation, updated based on KEGG and Uniprot databases 36, 50, 51.

b Function, pathway or biological role according to the KEGG and UniProt databases 50, 51.

c LC‐MS^E^ results, fold change, all genes *P* ≤ 0.01**,** boldfaced reached FDR control (BH) *q* ≤ 0.05.

d Predicted *in silico* by TargetRNA, TargetRNA2 or CopraRNA 56, 76, 77.

### Cytochrome *bc*_*1*_ levels are repressed upon expression of *nrrF*

**Figure 1:**
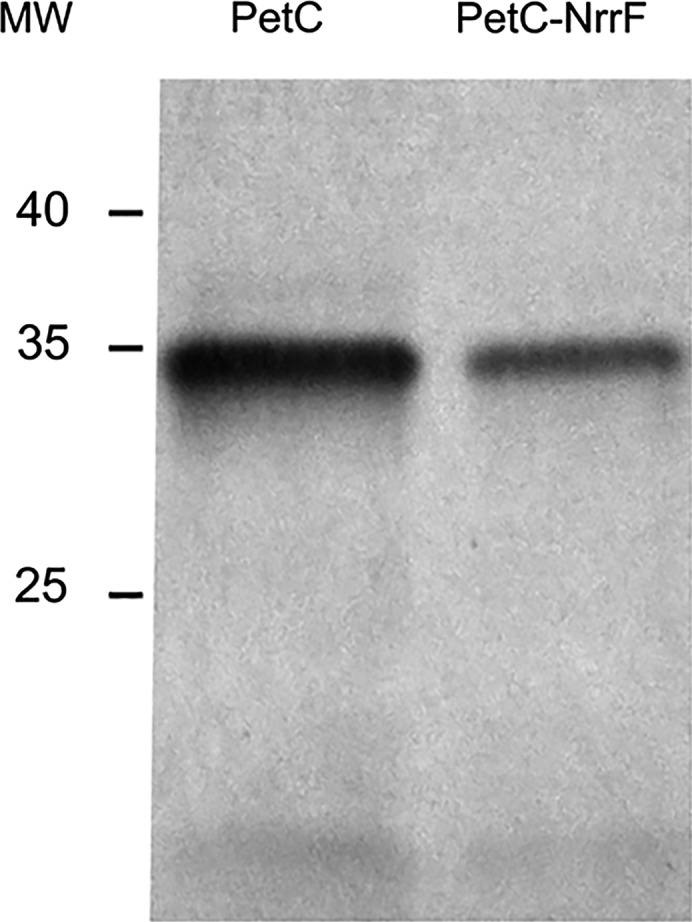
Cytochrome *bc*_1_ levels are repressed upon expression of *nrrF* in *Neisseria meningitidis*. FLAG‐stained protein blot showing altered expression of PetC in defined genetic backgrounds of meningococcal strain H44/76 before and after overexpression of *nrrF in trans*. Samples of total cell extracts of strain H44/76 carrying 3xFLAG‐tagged *petABC* before (lane PetC) and after overexpression of *nrrF* (lane PetC‐NrrF). Proteins were separated by SDS/PAGE, blotted and stained using an anti‐FLAG antibody. MW: molecular weight marker (kDa).

To confirm the mass proteomic result that expression of *petABC* was repressed upon expression of *nrrF*, we assessed protein levels of cytochrome *bc*_1_ before and after overexpression of *nrrF* in *N. meningitidis* H44/76. We engineered a *petABC::3XFLAG* construct and introduced it, by homologous recombination, in the *N. meningitidis* chromosome at the *petABC* locus. The recombinant was then transformed with a plasmid containing *nrrF* under control of an inducible promoter (pEN11_NrrF) to establish overexpression of *nrrF* independent of the iron concentration in the growth medium. Cytochrome *bc*_1_::3XFLAG levels were assessed before and after overexpression of *nrrF* by western blotting using anti‐FLAG antibodies. Relative transcript levels assessed by RT‐qPCR of NrrF are ~150‐fold higher after overexpression in meningococci, compared to levels in the wild‐type (wt) strain (relative transcript levels ~0.1) (not shown). Overexpression of *nrrF* reduced protein levels of cytochrome *bc*_1_::3xFLAG compared to corresponding levels in noninduced cells (~2.5‐fold; see Fig. 1). These results indicate that in meningococci, cytochrome *bc*_1_ levels are repressed upon expression of *nrrF*. This was confirmed by RT‐qPCR assessment of transcript levels of *petC*. Overexpression of *nrrF* reduced transcript levels of *petC* ~2‐fold (*P* < 0.0001) compared to levels in noninduced cells (not shown).

### *In silico* target interaction analysis of *petA*, a novel putative target of NrrF

**Figure 2:**
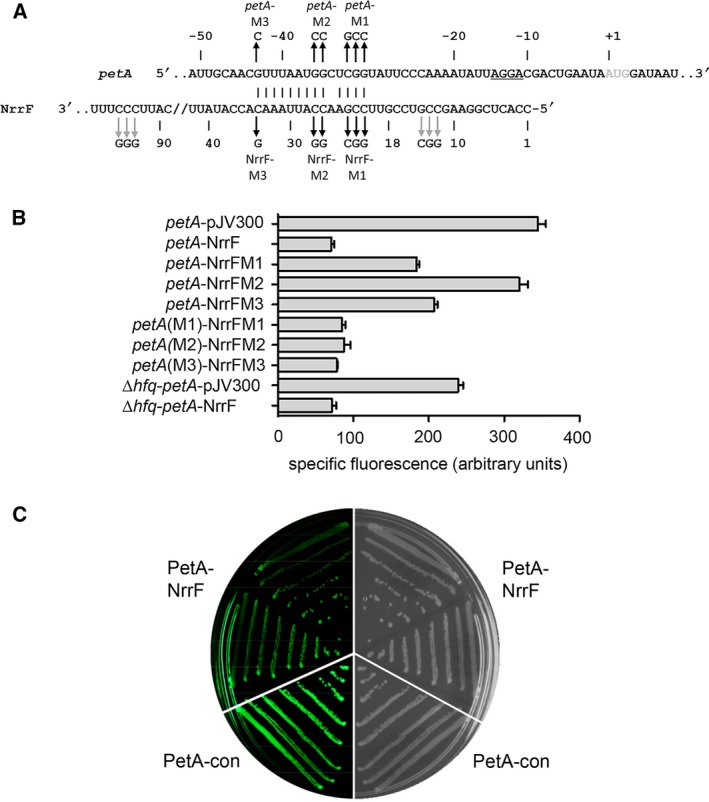
*In vitro* and *in vivo* analysis of the NrrF interaction with *petA* **.** (A) Schematic representation of the TargetRNA predicted base pairing interaction between NrrF and the 5′UTR of *petA*, supported by the RNAhybrid algorithm 55, 57. The +1 AUG initiation codon of *petA* is indicated in grey; the predicted SD sequence is underlined. Numbering of *petA* is relative to the A of the AUG codon; numbering of NrrF is relative to the transcriptional start site 37. Black arrows indicate complementary base pair changes (mutations yielding M1, M2 and M3 variants of NrrF or *petA*) introduced into NrrF expression and *petA::gfp* fusion plasmids. Grey arrows indicate base pair changes in NrrF outside the region of interaction. (B) NrrF and Hfq‐dependent regulation of assorted *petA::gfp* fusions *in vivo*. Specific fluorescence of cells expressing combinations of either wt target *petA::gfp* (*petA*) or mutant variants *petA*(M)*::gfp* fusion plasmids (*petA*(M1‐3)) in combination with sRNA control vector (pJV300) or plasmids expressing wt NrrF (NrrF) or mutant variants (NrrFM1–3) in the wt *Escherichia coli* background, or in the genetic background of a deletion *hfq*‐knockout strain (∆*hfq*). For details, see the main text. (C) Repression of translation fusion *petA::gfp in vivo*. Shown are images of an LB agar plate of *E. coli* carrying *petA::gfp* fusion plasmid in combination with control plasmid pJV300 (PetA‐con) or plasmid pNmNrrF expressing NrrF (PetA‐NrrF) obtained in the fluorescence mode (left panel) or the visible mode (right panel, mirrored image).

By using the computational tools TargetRNA and CopraRNA, a region of the 5′UTR of *petABC* (nucleotides −43 to −30; relative to the +1 AUG initiation codon of *petA*; A is +1) was predicted to base pair with nucleotide region 21 to 34 of NrrF 55, 56. The RNAhybrid algorithm confirmed this predicted NrrF‐*petA* duplex 57. The lowest free energy prediction (∆*G* = −21.8 kcal·mol^−1^) is schematically represented in Fig. 2A. Predicted base pairing of NrrF involves a 4 + 9‐bp duplex with the 5′UTR of the *petA* transcript. Nucleotides 21 to 34 of NrrF, except for nucleotide 25, form a perfect RNA duplex sequence with the 5′UTR of *petA* mRNA, spanning nucleotides −30 to −43, except for nucleotide −34. Interestingly, the predicted targeting region of NrrF does not seem to overlap with the SD or AUG sequence but is located upstream of these (16 and 30 nucleotides, respectively) (Fig. 2A). In addition, this region is extremely conserved among all meningococcal isolates and among all other (opportunistic) pathogenic *Neisseria* spp. (*Neisseria gonorrhoeae* and *Neisseria lactamica*) as present in the Bacterial Isolate Genome Sequence database (BIGSdb): 5348 (98%) of 5458 isolates of these three species carry a sequence identical to that found in strain H44/76 58. The same sequence is found in 18 strains of four commensal *Neisseria* spp. In all other commensal species (*n* = 7), represented by 86 isolates (2%), the locus is lacking or truncated.

### Translational repression of a *petA* 5′UTR containing region fused to *gfp* upon expression of *nrrF* in *Escherichia coli*

To investigate target recognition and translational control of *petABC* by NrrF, the direct interaction between the 5′UTR of *petABC* and NrrF was assessed *in vivo* using a well‐established green fluorescent protein (GFP) reporter system in which target mRNA fusions with GFP are co‐expressed with sRNA 28. Thus, the 5′UTR and AUG initiation codon region of *petA* (region −122 to +56) were fused in frame to *gfp* (*petA*::*gfp* fusion) in low copy vector pXG10 (recombinant plasmids are listed in Table 1); the resulting *petA::gfp* is constitutively transcribed from a P_LtetO‐1_ promoter (P_L_‐*gfp* expression) to specifically assay post‐transcriptional regulation. *Escherichia coli* cells transformed with the *petA*::*gfp* fusion were co‐transformed either with a plasmid expressing a nonsense (control) sRNA (pJV300) or with plasmid pNmNrrF expressing *nrrF*. Both these RNA are expressed from a constitutive P_LlacO‐1_ promoter 28, 59. *Escherichia coli* harbouring the plasmid pXG‐0 (*luciferase,* no *gfp*) transformed with pNmNrrF served as control for the effect of NrrF expression on autofluorescence of *E. coli*. In addition, cells containing the plasmid pXG‐1, expressing full‐length *gfp,* carrying a strong SD sequence and an artificial 5′UTR, were also co‐transformed with pJV300 and pNmNrrF to serve as controls for the aspecific effect of sRNA expression on P_L_‐*gfp* expression. Upon transformation, the level of expression of the *petA*::*gfp* fusion was assessed by quantifying GFP fluorescence activity of transformants in liquid culture. Autofluorescence of cells harbouring pXG‐0 and pNmNrrF or pJV300 was similar. In addition, fluorescence of cells carrying *gfp* (pXG‐1) with or without pNmNrrF or pJV300 was comparable, indicating that pNmNrrf expression has negligible effect on GFP fluorescence. However, cells co‐expressing *petA::gfp* with NrrF expressed significantly lower fluorescence compared to cells in which the *petA::gfp* fusion was co‐expressed with nonsense sRNA. Levels of *petA::gfp* were ~4‐fold repressed (*P* < 0.0001) upon co‐expressing NrrF compared to unregulated fluorescence levels [in the presence of a control nonsense RNA (pJV300)] (Fig. 2B,C). Reduced fluorescence of *petA::gfp* in the context of *nrrF* expression but not in the context of expression of a control nonsense sRNA confirms that NrrF represses the *petA* mRNA by a direct interaction between the 5′UTR of *petA* and NrrF, most likely via an antisense mechanism.

### NrrF represses petABC by a direct interaction between NrrF and the 5′UTR of *petABC*

To prove that predicted regions of NrrF and *petA* are indeed involved in base pairing and regulating expression, we engineered NrrF variants and introduced compensatory base pair exchanges in the target region of *petA* (depicted in Fig. 2A). The effect of the mutations on repression of *petA in vivo* was assessed using the *gfp*‐reporter system in *E. coli*. Nucleotides C_21_, C_22_ and G_23_ of *nrrF* were exchanged for G_21_, G_22_ and C_23_, respectively, to generate NrrFM1. Nucleotides C_26_ and C_27_ were exchanged for G_26_ and G_27,_ generating NrrFM2, and lastly, nucleotide C_34_ was exchanged for G_34_ to generate NrrFM3. Of note, transcript levels of NrrF variants were transcribed from the same constitutive promoter (P_LlacO‐1_) as in pNmNrrF and did not differ from levels of wt NrrF (not shown). Both NrrFM1 and NrrFM3 expression in the presence of wt target *petA*::*gfp* diminished repression (Fig. 2B). However, restoration of the predicted duplex by co‐expression of NrrFM1 and NrrFM3 with *petA*(M1)::*gfp* and *petA*(M3)::*gfp,* respectively (the latter containing compensatory base pair changes for the corresponding mutant NrrFs), restored repression to wt levels (Fig. 2B). These observations support the model of the predicted base pairing of NrrF to *petA*. Further proof for the direct interaction between NrrF and *petA* was obtained by the following experiments: Co‐expression of NrrFM2 in the presence of wt *petA*::*gfp* completely suppressed repression of *petA*::*gfp* (Fig. 2B). However, restoration of the predicted duplex by co‐expression of *petA*(M2)::*gfp*, containing the compensatory base pair mutations C_‐35_ and C_‐36_, completely restored repression to wt levels (Fig. 2B). Of note, transcripts of *petA* variants were transcribed from the same promoter (P_LtetO‐1_) as used for wt *petA* and were found at levels not different from those of wt *petA* (not shown). Mutations (C_12_C_13_G_14_→G_12_G_13_C_14_) or downstream (C_93_C_94_C_95_→G_93_G_94_G_95_) of NrrF predicted to interact with *petA* had no effect on regulation (not shown). Taken together, these results provide experimental validation for the predicted RNA duplex of NrrF with the 5′UTR of *petA* and confirm that NrrF regulates *petA* expression by an antisense mechanism resulting in repression of translation.

### Contribution of Hfq to post‐transcriptional regulation of *petA* by NrrF

The highly conserved protein Hfq has emerged as a key modulator of riboregulation 11, 60. Recently, we reappraised the meningococcal Hfq regulon, showing direct or indirect regulation of a large variety of cellular processes, including OMPs, the methyl citrate and TCA cycles, iron and zinc homeostasis and the assembly of ribosomal proteins 41. In another recent study, co‐immunoprecipitation combined with RNA sequencing of a FLAG‐tagged Hfq revealed an unexpectedly large Hfq‐RNA interactome, identifying 23 sRNA and 401 mRNA candidate targets 61.

To investigate the potential role of Hfq in regulation of *petA*, NrrF was first co‐expressed with *petA::gfp* in the *E. coli* Hfq‐knockout strain JVS‐2001. The amount of *nrrF* transcripts in JVS‐2001 was similar to that in wt cells (not shown). Of interest, specific fluorescence levels of cells expressing *petA*::*gfp* in the absence of NrrF expression were significantly lower (~1.4‐fold; *P* < 0.0001) in JVS‐2001 compared to those of wt (Hfq‐containing) cells expressing the *petA*::*gfp* fusion (Fig. 2B). Still, fluorescence of cells expressing *petA::gfp* was significantly downregulated (~3‐fold; *P* < 0.0001) upon NrrF expression (Fig. 2B). These observations indicate that Hfq is apparently not involved in the interaction between NrrF and *petA* mRNA in the cellular context of *E. coli*. Next, we tried to investigate whether repression of *petA* would also be found to be independent of Hfq in the genetic background of the meningococcus. Here, we used the previously engineered *petABC::3xFLAG* construct and introduced it in the chromosome at the *petABC* locus of ∆*hfq* 35. The amount of *nrrF* transcript levels in ∆*hfq petABC::3xFLAG* was similar to that in wt cells (not shown). Unfortunately, expression of *petABC::3xFLAG* in ∆*hfq* was below levels allowing quantification (data not shown).

## Discussion

Using proteomic analysis, we have shown that NrrF plays a role in the regulation of proteins involved in iron uptake, iron‐dependent metabolic processes and oxidative stress. Consequently, we analysed, to our knowledge for the first time, the *detailed* regulation of expression of *N. meningitidis* respiratory chain components by a sRNA. In addition, our results provide an explanation for the previously observed iron and Fur‐dependent expression of cytochrome *bc*_1_. Grifantini and colleagues showed higher transcript levels of *petC* in serogroup B meningococci grown in the presence of sufficient iron as compared to meningococci grown in the absence of iron 27. This was confirmed by Basler and colleagues 26 in serogroup C meningococci. In addition, transcript levels of *petA* were found to be induced by Fur 19. Here, we show that the iron and Fur‐regulated sRNA NrrF is ‘the missing link’, involved in the regulation of cytochrome *bc*_1_ by repressing *petABC* expression.

NrrF was the first meningococcal sRNA to be identified 21. It was shown that expression of *nrrF* is controlled by Fur and under iron‐depleted conditions, transcript levels of its putative target mRNA *sdhC* and *sdhA* were higher in a *nrrF*‐knockout mutant than in wt meningococci 21. It was further shown that NrrF forms a complex *in vitro* with a region of complementarity of the *sdhCDAB* transcript and this duplex formation most likely results in rapid turnover of the transcript 22. However, until now, *sdhCDAB* was the only target for NrrF which was experimentally validated.

We provide experimental evidence that cytochrome *bc*_1_, encoded by the polycistronic mRNA *petABC,* represents a novel target of NrrF. We showed that *petABC* expression is repressed by NrrF in meningococci. Next, we used a heterologous *gfp*‐reporter system to show that repression of *petABC* is the result of a direct interaction between NrrF and the 5′UTR of *petABC*, which was confirmed by (complementary) site‐directed mutagenesis of the regions of NrrF and *petABC* predicted to form a duplex. Surprisingly, we showed that this interaction seems to be independent of Hfq.

The region of NrrF interacting with *petABC* (nucleotides 21–34), as experimentally validated by site‐directed mutagenesis in this study, is of interest for several reasons. First, this region is part of the region of NrrF (nucleotides 19–86) that is suggested to also interact with the intergenic region between *sdhD* and *sdhA* of the polycistronic mRNA *sdhCDAB* 21, 22. Unfortunately, in none of these studies, this *in silico* observation was further experimentally validated or delineated. Our data suggest that a region of NrrF as short as 14 nucleotides, composed of two stretches of 4 and 9 Watson–Crick pairs, is sufficient to control regulation of *petA*. Relative short regions of interactions have been described before. For example, only six bps are critical in SgrS‐PtsG 5 and RybB duplexes with OMP mRNA range from 15 to 8 bp 62. Our observation that regulation of *petA* is completely suppressed in NrrFM2 (having 2 C→G mutations at positions 26 and 27) but completely restored by compensatory substitutions in the 5′UTR of petA(M2) (having 2 G→C mutations at positions −35 and −36) provides ultimate proof for the direct interaction of NrrF and the 5′UTR of *petA* and for the fact that this interaction is essential for regulation.

We found the experimentally validated region of interaction, contrary to the flanking nucleotides up‐ and downstream of it, to be extremely well conserved among the pathogenic meningococci and gonococci, and in the opportunistic pathogen *N. lactamica*. In commensal *Neisseria* spp., this region of interaction is largely truncated or absent. Differences in the site of interaction in *nrrF* between *Neisseria* spp. may reflect differences in abilities to fine‐tune responses to fluctuating iron availability during the specific environmental challenges they encounter.

Another interesting feature of this interaction is the localization of the duplex. It should be noted that many of the sRNA analysed to date inhibit translational initiation of mRNA targets by sequestering the SD and/or AUG sequence of the RBS and thus act close to these sequences 63. Duplex formation leading to translational interference was proposed to serve as the primary mechanism of target repression, irrespective of concomitant mRNA degradation 64. However, some sRNA regulate their targets by alternative mechanisms that involve base pairing more upstream of the SD or more downstream of the AUG sequences. This mode of action might exclude a mechanism that relies on direct competition with initiating ribosomes and suggests that the mRNA window for target repression could be broader than the RBS. Here, we showed that duplex formation between NrrF and *petA* encompasses the region −43 to −30 of *petABC*, apparently not on top of the SD and/or AUG sequence of the RBS. However, the physical boundaries of the RBS extend from nucleotides −35 to +19 relative to AUG (A is +1) 65, 66. This means that duplex formation between NrrF and *petA* could take place just inside the physical boundaries of the RBS, possibly resulting in translational interference.

In numerous Gram‐negative bacteria, the RNA‐binding protein Hfq assists in duplex formations between sRNA and target mRNA by enhancing local RNA concentrations, changing RNA structures and accelerating strand exchange and annealing 11. Although many *trans*‐acting sRNA characterized require Hfq for base pairing, there are exceptions, among which sRNA in *Vibrio cholerae* as well as in *E. coli* 67, 68. When we investigated the involvement of Hfq in the regulation of *petA*, we found that in the *E. coli* reporter system, NrrF‐mediated repression of *petA* is independent of Hfq. It should be noted that this might be because *E. coli* Hfq cannot efficiently interact with *N. meningitidis* NrrF due to structural differences between *E. coli* and meningococcal Hfq. However, amino acid homologies of *E. coli* Hfq and *N. meningitidis* Hfq are comparable to those of Hfq of *E. coli*,* V. cholerae* and *Salmonella enterica* serovar Typhimurium 35. Furthermore, *N. meningitidis* Hfq has been shown to target endogenous *Salmonella* sRNA, providing evidence of a conserved inherent sRNA‐binding property of Hfq 69. Small RNA·mRNA interactions of the latter two spp. have been successfully assessed in the *gfp*‐reporter system used here and confirmed in the genetic background of the spp. themselves 28. Previously, the interaction between NrrF and *sdhC* has been shown to be Hfq independent as well, and there is no *in vivo* experimental evidence showing SdhC to be part of the Hfq regulon 41, 70. An alternative explanation for the observed lack of Hfq dependency is illustrated by the behaviour of sRNA DsrA. In an *E. coli hfq* mutant, chromosome‐expressed DsrA was unstable. When expressed from a multicopy plasmid, DsrA was stable in both wt and *hfq* mutant strains, but it had only partial activity in the *hfq* mutant strain 71. This might also explain the lacking Hfq dependency observed for NrrF‐mediated regulation in *E. coli*, as NrrF is expressed from a constitutive promoter in a multicopy plasmid. In the *E. coli* system, NrrF‐mediated repression of *petA* was the same in wt and in ∆*hfq* cells, although we noted that *petA* expression in the *E. coli hfq* mutant was significantly lower (*P* < 0.001) compared to *petA* expression levels in wt cells. Taking this into account, these results suggest that Hfq itself is involved in the stability of this transcript, irrespective of NrrF levels or duplex formation between NrrF and *petA*. The exact mechanism by which the duplex formation between NrrF and the 5′UTR of *petABC* leads to translational repression awaits further experimentation. Our transcript analyses and target protein determinations in meningococci are compatible with a mechanism in which NrrF allows degradation of *petABC*. Such sRNA‐mediated degradation of targets has been described for other sRNA such as MicM in concert with its target *ybfM* and for Qrr3 in concert with *luxO* 13, 72, 73.

The importance of concomitant regulation of the Krebs cycle and the respiratory chain cannot be overstated. They are linked both directly (succinate dehydrogenase being part of both the Krebs cycle and the respiratory chain) and indirectly (the respiratory chain oxidizing, and thus recycling, the electron‐rich compounds NADH and FADH_2_ formed by the Krebs cycle). Previously, we have shown that the sibling sRNA NmsRs target genes encoding TCA cycle enzymes including SdhC, which is a target of NrrF as well. It is therefore plausible that regulation on the level of translation of proteins involved in both pathways is tightly coordinated by at least two sRNA.

All known cytochrome *bc*_1_ complexes are integral membrane modules, varying wildly in subunit composition. In meningococci, the cytochrome *bc*_1_ complex consists of only three subunits, cyt *b* (PetB), cyt *c*_1_ (PetC) and the iron‐sulfur protein (ISP) (PetA) 31, 74, all of them essential in coupling electron transfer to proton translocation over the plasma membrane. In this way, chemical energy is converted into a membrane potential (a proton gradient) which can be used for ATP synthesis. The three subunits all use iron as an essential cofactor in their prosthetic groups. Cyt *b* has two b‐type haemes (low potential *b* haeme, *b*_L_, as well as high potential *b* haeme, *b*_H_), cyt *c*_1_ has a single c‐type haeme, and ISP contains a two‐iron–two‐sulfur (2Fe‐2S) cluster. The complex thus nicely illustrates the vital role of iron in bacterial metabolism. At the same time, free iron is toxic (e.g. contributing to ROS formation) and the cellular response to iron levels in the environment should be fine‐tuned. We show NrrF to be exquisitely involved in this regulation.

## Author contributions

YP, RHV, DS and AvdE conceived and designed the project. RHV, KS, SB and GK acquired the data. YP, RHV, KS, SB, GK, DS and AvdE analysed and interpreted the data. YP, RHV, DS and AvdE wrote the manuscript.

## Supporting information


**Table S1.** Oligonucleotides used in this study.

**Table S2.** Differential protein profiling (LC‐MS^E^) results of *N. meningitidis* H44/76Δ*nrrF*+pEN11_Empty and H44/76Δ*nrrF*+pEN11_*nrrF*.
